# Patient-reported outcomes in venous leg ulcers: results from a prospective multicenter single-arm cohort study of silicone superabsorbent polymer dressings

**DOI:** 10.1080/1179271X.2026.2734965

**Published:** 2026-10-04

**Authors:** Sebastian Probst, Vladica Veličković, Jacek Mikosinski, Konrad Panczak, Adam Wegrzynowski, Dorota Piechota, Marek Kotala, Przemyslaw Lipinski, Katarzyna Rybolowicz, Agnieszka Lipinska, Jacek Bil, Teresa Szenk, Patrycja Buczak, Cornelia Santoso, Anitha Pitchika, David G. Armstrong

**Affiliations:** a Geneva School of Health Science, HES-SO, Switzerland; b Care Directorate, Geneva University Hospitals, Switzerland; c Medical Faculty, University of Geneva, Switzerland; d Faculty of Medicine, Nursing and Health Sciences, Monash University, Australia; e College of Medicine, Nursing and Health Sciences, University of Galway, Ireland; f Evidence Generation Department, HARTMANN GROUP, Germany; g Institute of Public Health, Medical Decision Making and HTA, UMIT, Austria; h General and Vascular Surgery, Mikomed Sp. z o.o., Poland; i General and Vascular Surgery, LECRAN—Centrum Opieki nad Ranami-Kunickiego, Poland; j Internal Medicine, Podovia Sp. z o.o., Poland; k Internal Medicine, JBS Klinika Sp. z o.o., Poland; l Vascular Surgery, MelissaMed Poradnia Chirurgiczna, Poland; m General Surgery, NZOZ “ARGO” Centrum Medyczne, Poland; n General Surgery, Specjalistyczny Ośrodek Leczniczo-Badawczy, Poland; o Department of Cardiology, National Medical Institute of the Ministry of Warsaw and Administration, Poland; p Statistics Department, Clean Data Labs, Poland; q Clinical Operations, Sphera Clinical Research, Poland; r Keck School of Medicine, University of Southern California, USA

**Keywords:** Venous leg ulcer, patient-reported outcomes, quality of life, silicone superabsorbent dressing, outcomes research, wound-QoL, patient benefit index

## Abstract

**Background:**

Venous leg ulcers (VLUs) are chronic wounds associated with substantial symptom burden, impaired health-related quality of life, and high healthcare costs. Current guidance recommends assessing patient-centered outcomes alongside healing. This study reports the VLU subgroup of a prospective multicenter cohort, focusing on wound-related quality of life and patient-perceived treatment benefit.

**Methods:**

This prospective, single-arm cohort study was conducted in specialist wound centers in Poland. Adults with chronic, noninfected, exuding VLUs received RespoSorb Silicone Border or RespoSorb Silicone dressings together with standardized compression therapy for up to 6 weeks. Patient-reported outcomes (PRO) were the Wound Quality of Life questionnaire (Wound-QoL-17) and the Patient Benefit Index for wounds (PBI-W). Changes in Wound-QoL from baseline to final visit, responder analyses using the published 0.50-point minimal important difference and attained PBI-W scores at follow-up were analyzed.

**Results:**

Fifty-two patients with VLU were included (44.2% women; mean age 69.1 years). Final PRO data were available for 48 patients. Mean global Wound-QoL improved from 2.03 at baseline to 1.18 at final visit (mean change 0.81; 95% CI 0.49–1.12; *p* < 0.001; standardized response mean 0.74). Clinically meaningful improvement (≥0.50 points) occurred in 56.3% of patients. Final attained PBI-W benefit was high (mean 2.92 ± 0.97), with 93.8% achieving PBI-W ≥ 1 and 58.3% achieving PBI-W ≥ 3. No dressing-related serious adverse events were reported.

**Conclusions:**

Silicone superabsorbent polymer dressings used with compression were associated with improved quality of life and high patient-perceived benefit. Findings support incorporating validated patient-reported outcomes in VLU research and warrant larger comparative studies.

**Trial Registration:**

ClinicalTrials.gov Identifier NCT07549737 (Date of registration: 24.04.2026). Registration was completed retrospectively due to an administrative oversight during a transition in study leadership. The study protocol and prespecified study procedures were established before enrollment began, and the statistical analysis plan was finalized before database lock. No changes were made to the study objectives, endpoints, eligibility criteria, conduct, statistical analyses, or reporting as a result of the delayed registration.

## ARTICLE HIGHLIGHTS

- This study reports the VLU subgroup of a prospective multicenter single-arm cohort study of silicone superabsorbent polymer dressings used under compression.
- Final/discontinuation PRO data were available for 48 of 52 patients; no ad hoc imputation was used for missing visit-level PRO data.
- Global Wound-QoL improved by 0.81 points (95% CI 0.49 to 1.12), and 56.3% of patients achieved the prespecified ≥0.50-point threshold for clinically relevant improvement.
- Final attained PBI-W benefit was high with a mean of 2.92 ± 0.97 and reduced Wound-QoL burden was associated with greater patient-perceived treatment benefit.

## Introduction

Venous leg ulcers (VLUs) are among the most severe and resource-intensive manifestations of chronic venous insufficiency and represent a major subgroup of chronic lower-extremity ulcers. A recent systematic review and meta-analysis reported a pooled VLU prevalence of 0.32% and pooled incidence of 0.17%, with substantial heterogeneity across studies.^1^ Health-economic estimates also vary by data source and case ascertainment. In one UK routine-data analysis, direct annual management costs were estimated at £7706 per patient, corresponding to nearly £2 billion nationally, with community nursing visits identified as the principal cost driver.^2^ A later English cross-sectional study reported a lower annual estimate of £4787.70 per person and £102 million nationally, but likewise found that community staff time represented more than half of short-term treatment costs.^3^ Beyond delayed healing and recurrence, patients experience pain, exudate leakage, malodour, sleep disturbance, impaired mobility, psychological distress, and social restriction, all of which negatively affect health-related quality of life (HRQoL) in systematic reviews of leg ulceration and chronic VLU burden.^4^^,^^5^

Contemporary European and international guidance frames VLU management as both local wound care and vascular disease management. The European Society for Vascular Surgery (ESVS) guideline recommends compression therapy for active venous ulceration and treatment of incompetent superficial veins in patients with active or healed venous ulceration to accelerate healing and reduce recurrence.^6^ The German S2k guideline on VLU similarly emphasizes structured diagnosis, vascular assessment, color-coded duplex sonography, compression, and treatment of superficial venous insufficiency when indicated.^7^ International recommendations from the Society for Vascular Surgery/American Venous Forum and the European Wound Management Association likewise emphasize comprehensive assessment, arterial screening with ankle-brachial pressure index, duplex ultrasound, compression, wound-bed management, and attention to factors that influence adherence and implementation.^8^^,^^9^ A 2024 evidence map of 19 VLU clinical practice guidelines further showed that assessment recommendations increasingly include pain, psychosocial status, QoL, and disease-specific outcome assessment alongside traditional vascular and wound parameters.^10^

These clinical and economic consequences make VLUs highly relevant to outcomes research. However, outcome reporting remains heterogeneous, with inconsistent inclusion of patient-reported domains.^11^ The subsequent international eDelphi core outcome set concluded that healing, pain, quality of life, resource use, and adverse events should be reported in all randomized evaluations of interventions for venous leg ulceration.^12^ This has direct implications for dressing studies, in which performance is frequently described in terms of exudate handling, dressing-change frequency, or wound area reduction, while the patient perspective may be less completely characterized. Accordingly, evaluating patient-reported outcome measures (PROM)s alongside clinical outcomes is essential for assessing treatment value.

The choice of PROMs also requires explicit justification. Systematic reviews of HRQoL and PROM instruments in VLU have found that measurement-property evidence remains limited and heterogeneous; earlier work identified the Nottingham Health Profile and VLU Quality of Life (VLU-QoL) as the most suitable available instruments, while later COSMIN-informed review work provisionally recommended VLU-QoL but emphasized the need for further validation in active VLU populations.^13–15^ Wound Quality of Life (Wound-QoL) offers a pragmatic chronic-wound-specific alternative because it was developed from three established HRQoL instruments - the Freiburg Life Quality Assessment for wounds, Cardiff Wound Impact Schedule, and Würzburg Wound Score - and was originally derived in a leg ulcer population.^16^ Subsequent work supported its validity, feasibility, reliability, responsiveness, and content validity in chronic wounds, including leg ulcers.^17^^,^^18^ The Patient Benefit Index for wounds (PBI-W) provides complementary information by assessing treatment benefit in relation to individually weighted patient goals, and it has been developed and used specifically for chronic wounds and outpatient leg-ulcer care.^19^^,^^20^ Together, these instruments capture both wound-related burden and patient-perceived benefit.

Effective exudate management provides the mechanistic rationale for silicone superabsorbent polymer (SAP) dressings in VLU care. International consensus guidance highlights that exudate can delay healing and cause complications when present in the wrong amount, location, or composition, making systematic assessment and management important for preventing maceration, leakage, and other exudate-related harms.^21^ SAP dressings are intended to manage moderate-to-high exudate, protect peri-wound skin, and reduce leakage and dressing-change burden. Their design combines a soft silicone wound-contact layer that supports atraumatic removal with a highly absorbent SAP core capable of absorbing and retaining substantial volumes of exudate, including protease-rich wound fluid, even under compression. By maintaining moisture balance, limiting peri-wound maceration, and reducing exudate leakage, SAP dressings may help create a more favorable wound environment while improving patient comfort and overall treatment experience. A 2024 systematic review of clinical and economic evidence for superabsorbent wound dressings found signals of reduced dressing-change frequency, lower pain scores, and possible cost-effectiveness compared with standard dressings, while also concluding that the evidence base remains limited because most included clinical studies were case series or otherwise noncomparative.^22^ Observational studies of silicone or siliconized SAP dressings have reported favorable exudate handling and patient or clinician assessments, although evidence remains limited.^23^^,^^24^

The main publication of the clinical study reported clinically meaningful wound outcomes in chronic VLUs and diabetic foot ulcers treated with silicone SAP dressings in a prospective single-arm clinical study.^25^ The present study builds on that main study by focusing specifically on the VLU subgroup and by foregrounding wound-related quality of life and patient-perceived treatment benefit. The objective was to describe wound trajectory, Wound-QoL, PBI-W, and safety outcomes in patients with VLUs treated with silicone SAP dressings under compression therapy.

## Methods

### Study design, setting, and reporting approach

This analysis reports a predefined VLU subgroup from a prospective, single-arm, multicenter cohort study evaluating silicone superabsorbent polymer dressings in chronic wounds. Recruitment was conducted between July 2024 and August 2025 in eight specialized wound care centers in Poland. This study was conducted in accordance with the principles of the Declaration of Helsinki. The study protocol, informed consent form, case report forms, insurance conditions, and related study documents were approved by the Independent Ethics Committee of the Lower Silesian Medical Chamber in Wroclaw (Komisja Bioetyczna przy Dolnośląskiej Izbie Lekarskiej we Wrocławiu) on May 8, 2024 (approval No. 03/05/2024), based on protocol version 1.0 dated March 26, 2024. All participants provided written informed consent before study-specific procedures. This study is registered at ClinicalTrials.gov (Identifier: NCT07549737; Date of registration: 24.04.2026).

Participants were followed for up to 6 weeks or until complete healing, withdrawal, or discontinuation of dressing use if no longer clinically indicated. The 6-week observation period was predefined in the parent study protocol and was selected to evaluate short-term clinical outcomes, including wound area reduction and patient-reported outcomes during active treatment. Early wound area reduction has been consistently shown to predict subsequent healing in chronic ulcers, with larger reductions in wound size being associated with progressively greater probabilities of eventual wound closure. Study visits were conducted according to a predefined schedule with weekly assessments; Visit 1 represents baseline and visit 7 reflects the end-of-follow-up assessment within the planned observation period of 6 weeks.

The study was prepared in accordance with the Transparent Reporting of Evaluations with Nonrandomized Designs (TREND) statement for nonrandomized evaluations and with CONSORT-PRO reporting principles for patient-reported outcomes (PROs) where applicable to a non-randomized design.^26^^,^^27^ PRO selection, analysis, and reporting followed established methodological and regulatory guidance.^28–35^

### Participants

Eligible participants were adults aged 18 years or older with a chronic, exuding VLU of 1 to 12 months’ duration, clinically diagnosed by the treating physician and considered suitable for conservative wound management and standardized photography. Vascular eligibility was confirmed by an ankle-brachial pressure index between 0.8 and 1.2: in case where ABPI was > 1.2, toe-brachial pressure index was assessed and required to be >0.6. Ulcers were required to be noninfected at inclusion and confined to full-thickness skin involvement without exposure of tendon, muscle, or bone.

Key exclusion criteria included hypersensitivity to study product components; more than two full-thickness ulcers on one limb or more than three ulcers in total; recurrent ulcers; hypertensive leg ulcers; NYHA class IV heart failure; severe deep vein thrombosis or contraindications to compression therapy; clinical infection requiring antimicrobial treatment; significant ischemia or clinically significant intermittent claudication; osteomyelitis; uncontrolled edema; uncontrolled diabetes (HbA1c ≥8.6%); severe malnutrition (albumin <3.5 g/dL); severe obesity (BMI >40 kg/m^2^); immunosuppressive therapy; recent use of topical corticosteroids, hyperbaric oxygen therapy, or bioengineered tissue products; malignant wounds; pregnancy or breastfeeding; inadequate contraception; and any condition that could compromise reliable wound assessment or study participation.

### Intervention and concomitant care

Participants received either RespoSorb Silicone Border (also marketed as Zetuvit Plus Silicone Border, PAUL HARTMANN AG, Germany) or RespoSorb Silicone (also marketed as Zetuvit Plus Silicone, PAUL HARTMANN AG, Germany), selected according to wound characteristics and routine clinical judgement (non-standardized allocation). Both dressings are silicone-coated superabsorbent polymer products designed for high exudate handling and atraumatic removal. The dressings feature a multilayer structure designed to support effective exudate management and peri-wound skin protection. This includes a perforated silicone wound-contact layer to facilitate atraumatic removal, a hydrophilic distribution layer that promotes even fluid distribution, a cellulose-based SAP core with high fluid retention capacity, and a breathable backing layer that permits gas exchange while reducing the risk of external contamination. The bordered version additionally incorporates an adhesive polyurethane film that secures the dressing without secondary fixation and provides shower-resistant protection, whereas the non-bordered version requires fixation according to routine clinical practice.^24^ All VLU participants received standardized compression therapy throughout follow-up, consistent with evidence supporting compression as the foundation of conservative VLU treatment.^36^ Patients requiring antimicrobial therapy or receiving advanced wound-healing interventions, as specified in the exclusion criteria, were not eligible for study participation. Wound photographs were obtained before and after cleansing/debridement at each scheduled visit, and dressing changes were documented in the electronic case report form. Management of comorbidities was not protocol-directed and remained at the discretion of the treating physicians according to routine clinical practice.

### Outcome measures

Clinical effectiveness outcomes included wound area reduction over time and complete wound healing by visit. Wound area was assessed by centralized digital planimetry based on standardized wound photographs obtained before and after cleansing/debridement; photodigital planimetry has been validated for wound measurement and rater reliability.^37^

The VLU-specific outcomes research focus of this study was on patient-reported outcomes. The PROM objective was to describe wound-related quality-of-life burden over time and patient-perceived treatment benefit associated with silicone superabsorbent dressings used under compression. This distinction was prespecified conceptually because Wound-QoL-17 and PBI-W measure different constructs: Wound-QoL-17 assesses longitudinal wound-related impairment, whereas PBI-W captures patient-defined treatment benefit relative to individual treatment goals.

Wound-related quality of life was assessed using the 17-item Wound-QoL questionnaire, a chronic-wound-specific HRQoL instrument developed from three established disease-specific chronic-wound instruments and subsequently validated for feasibility, reliability, validity, and responsiveness in chronic wound populations.^16–18^ The questionnaire assesses impairment during the preceding 7 days. Item responses are scored from 0 (not at all) to 4 (very much), with lower scores indicating less impairment. The global score was calculated as the mean of all valid items when at least 13 of 17 items were completed; body, psyche, and everyday-life subscales were calculated according to the developer manual, with no more than one missing item permitted per subscale.^38^ The Wound-QoL manual documents a linguistically validated Polish version, consistent with the study setting.^16^ For interpretability, changes of approximately 0.50 points or greater in the Wound-QoL global score were considered patient-relevant based on published minimal important difference estimates.^39^

Patient-perceived treatment benefit was assessed using the Patient Benefit Index for wounds (PBI-W), which was developed and validated to measure patient-relevant benefit in chronic wound therapy.^19^ PBI-W is not a conventional baseline-to-follow-up symptom change score. It is a weighted benefit index derived from pre-treatment ratings of treatment needs and post-treatment ratings of the extent to which these goals were achieved, resulting in a 0 to 4 score in which higher values indicate greater patient-relevant benefit.^19^ A pilot study in leg-ulcer outpatients supports the feasibility of this patient-defined benefit approach in this clinical context.^20^ Accordingly, PBI-W was summarized primarily as an attained treatment-benefit endpoint at follow-up/final visit, rather than as a conventional longitudinal change endpoint.

Perceived social support was characterized at baseline using the Multidimensional Scale of Perceived Social Support (MSPSS), a validated self-report measure of perceived support from family, friends, and significant others.^40^ MSPSS was included as a contextual factor because it may influence patient-reported symptom burden, coping, and treatment experience. Safety outcomes included adverse events and dressing-related serious adverse events.

### Statistical analysis

Analyses were conducted in the full analysis set, defined as all eligible participants who received at least one application of a study dressing and contributed at least one post-baseline assessment. This terminology was used instead of intention-to-treat because the intention-to-treat principle is conventionally tied to randomized trial settings in ICH E9, whereas the present analysis is a nonrandomized single-arm cohort.^41^

Continuous variables were summarized as means with standard deviations or medians with interquartile ranges, and categorical variables as counts and percentages. The final visit for PRO analysis was defined as Visit 7 or if earlier, last visit up to complete healing or discontinuation/withdrawal. No formal sample size calculation was performed for this analysis, as it represents a subgroup analysis of a parent study with a predefined sample size. The parent study was powered for its primary endpoint on wound area reduction, with a planned sample size of at least 80 evaluable patients providing 80% power to demonstrate superiority over a predefined benchmark for wound size reduction. The present VLU analysis represents an exploratory subgroup analysis and was not separately powered for PRO endpoints or subgroup comparisons; therefore, all *p*-values were interpreted descriptively, and no multiplicity adjustment was applied.

Wound-QoL-17 was treated as the longitudinal patient-reported burden endpoint. The primary exploratory analysis estimated within-person change in Wound-QoL-17 global and subscale scores from baseline to final visit. Mean change was reported with 95% confidence intervals. Paired t-tests were used for the main within-person comparisons when change scores were approximately normally distributed, and Wilcoxon signed-rank tests were used as nonparametric sensitivity analyses. Standardized response metrics, including the standardized response mean where appropriate, were used to support interpretation of responsiveness, consistent with recommendations to evaluate PRO change using magnitude, precision, and clinical meaningfulness rather than relying on *p*-values alone.^39^^,^^42^

Patient-level responder analyses were performed for the Wound-QoL-17 global score using the published minimal important difference of approximately 0.50 points.^39^ Participants were classified as improved when the global score decreased by at least 0.50 points, worsened when it increased by at least 0.50 points, and unchanged when the absolute change was less than 0.50 points. Responder proportions were summarized descriptively.

PBI-W was analyzed descriptively as a weighted attained treatment-benefit score at follow-up/final visit. Baseline patient-need ratings and follow-up benefit ratings were treated as components of the PBI-W framework rather than as directly comparable repeated measures, and no primary inferential baseline-to-final change test was specified for PBI-W.^19^^,^^20^ Final PBI-W scores were summarized using mean, standard deviation, 95% confidence interval, median, and interquartile range. Proportions in descriptive score bands (≥1, ≥2, and ≥3) were used to characterize the distribution of attained patient-relevant benefit; these bands were not treated as validated responder thresholds.

Longitudinal mixed-effects models were not used as primary analyses for the PRO endpoints because the PROM assessment structure in this subgroup was limited to baseline and final/discontinuation assessments for Wound-QoL-17 and to pre-treatment needs plus follow-up benefit ratings for PBI-W. Contemporary PRO analysis guidance supports mixed-effects models when the research objective is to estimate longitudinal change over multiple post-baseline PRO assessments and to use all available repeated observations; however, such a model did not align with the defined PRO objective or the available PROM time structure for this subgroup.^43^

Item-level missing data were handled according to each instrument’s scoring manual; no ad hoc item imputation was used beyond manual-specified scoring rules. Visit-level missing PRO data and reasons for missingness, including healing, withdrawal, and discontinuation of dressing use, were summarized descriptively. This approach is consistent with ICH E9, EMA missing-data guidance, and PRO-specific missing-data recommendations emphasizing prospective prevention of missingness, transparent reporting, and avoidance of unjustified last-observation-carried-forward or other ad hoc imputation procedures.^41^^,^^44–46^

All statistical analyses were performed using R version 4.1.3 (R Foundation for Statistical Computing, Austria) or later.

## Results

### Participant characteristics

A total of 86 patients with chronic wounds were screened for participation. Of these, 85 entered the safety population and 80 met the criteria for inclusion in the full analysis set of the parent study population. Twenty-eight patients had diabetic foot ulcers and were not included in the present subgroup analysis. The VLU subgroup comprised 52 patients (Figure 1), including 34 treated with RespoSorb Silicone Border and 18 treated with RespoSorb Silicone. Women represented 44.2% of the cohort. Mean age was 69.1 ± 13.6 years and mean body mass index was 30.00 ± 5.64 kg/m^2^. Hypertension was present in 63.5% of patients, obesity in 40.4%, diabetes in 19.2%, deep vein thrombosis in 13.5%, and previous venous surgery in 23.1%. Mean ABPI was 1.02 ± 0.13. Ulcers were longstanding, with a median duration of 180.5 days (IQR 98.0–278.0), and were located mainly on the calf or ankle. Baseline characteristics are summarized in Table 1. Lifestyle and prior wound treatment before enrolment are summarized in Supplementary Table S1.

**Figure 1.**
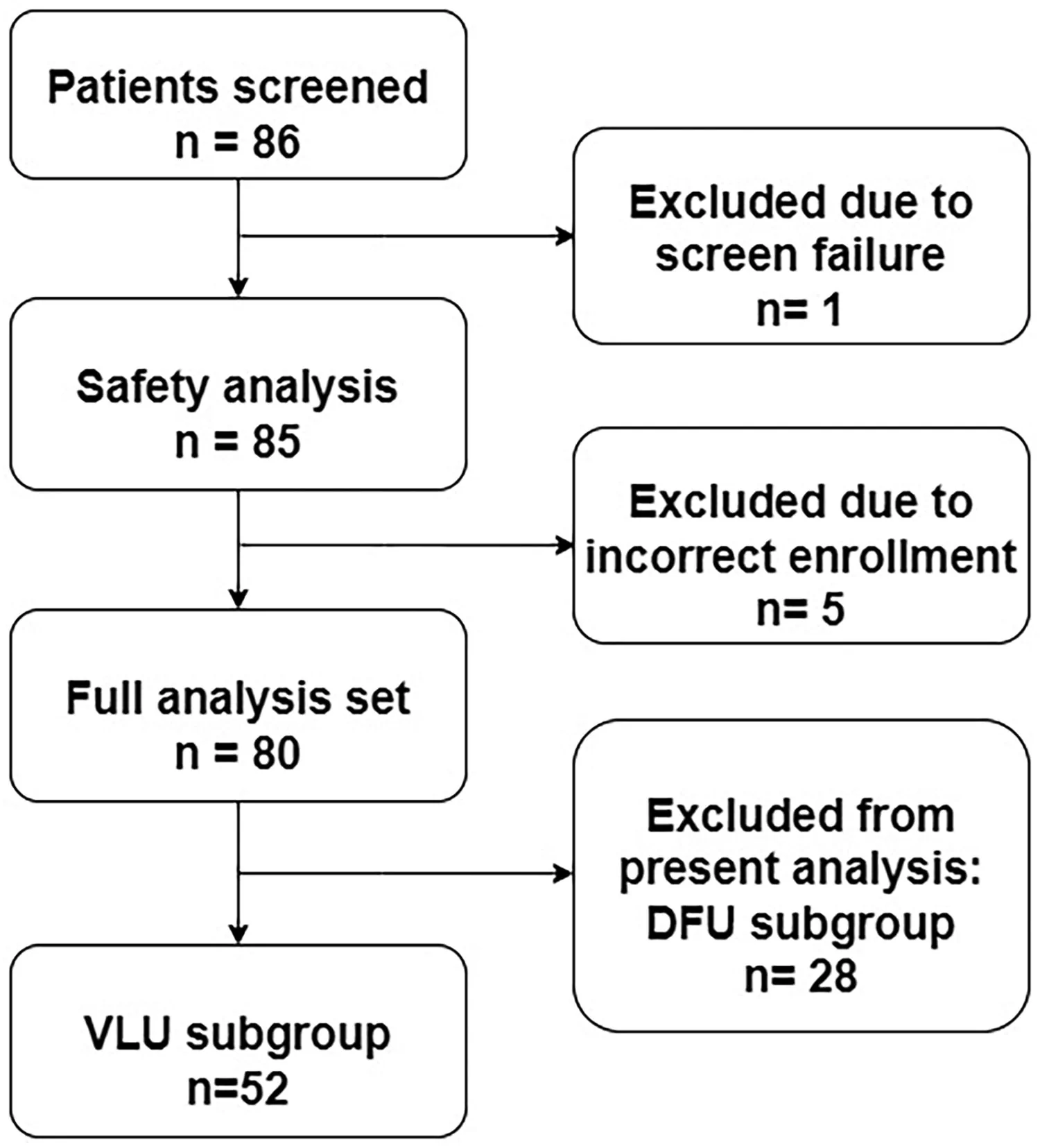
Flow diagram of participant selection and derivation of the venous leg ulcer (VLU) subgroup.

**Table 1.** Baseline characteristics of the VLU subgroup.

Characteristic	Overall (*N* = 52)	RSSB (*n* = 34)	RSSil (*n* = 18)
Female, *n* (%)	23 (44.2)	17 (50.0)	6 (33.3)
Age, years, mean (SD)	69.1 (13.6)	68.0 (14.1)	71.2 (12.8)
BMI, kg/m², mean (SD)	30.00 (5.64)	29.73 (5.72)	30.52 (5.63)
Hypertension, *n* (%)	33 (63.5)	21 (61.8)	12 (66.7)
Diabetes, *n* (%)	10 (19.2)	5 (14.7)	5 (27.8)
Obesity, *n* (%)	21 (40.4)	13 (38.2)	8 (44.4)
Deep vein thrombosis, *n* (%)	7 (13.5)	5 (14.7)	2 (11.1)
Previous venous surgery, *n* (%)	12 (23.1)	6 (17.6)	6 (33.3)
ABPI, mean (SD)	1.02 (0.13)	0.99 (0.12)	1.08 (0.13)
Ulcer duration, days, median (IQR)	180.5 (98.0, 278.0)	181.5 (96.0, 286.0)	156.5 (101.0, 269.0)
Location: calf, *n* (%)	32 (61.5)	19 (55.9)	13 (72.2)
Location: ankle, *n* (%)	19 (36.5)	14 (41.2)	5 (27.8)

Values are presented as number (%) or mean (SD), except ulcer duration, which is shown as median (IQR).

Abbreviations. ABPI, ankle-brachial pressure index; BMI, body mass index; RSSB, RespoSorb Silicone Border; RSSil, RespoSorb Silicone.

### Wound healing outcomes

Wound area reduction improved progressively across follow-up in the VLU subgroup. Based on centralized summary plots, mean reduction increased from 8.6% at Visit 2 to 51.2% at Visit 7 before cleansing/debridement, and from 13.8% to 55.0% after cleansing/debridement. Complete healing was not observed at Visits 2 through 6, but by Visit 7, 15 of 51 evaluable ulcers (29.4%) were documented as completely healed overall, including 8 of 33 (24.2%) in the bordered-dressing subgroup and 7 of 18 (38.9%) in the non-bordered subgroup. Figure 2 summarizes wound area reduction trajectories before (Figure 2A) and after cleansing/debridement (Figure 2B).

**Figure 2.**
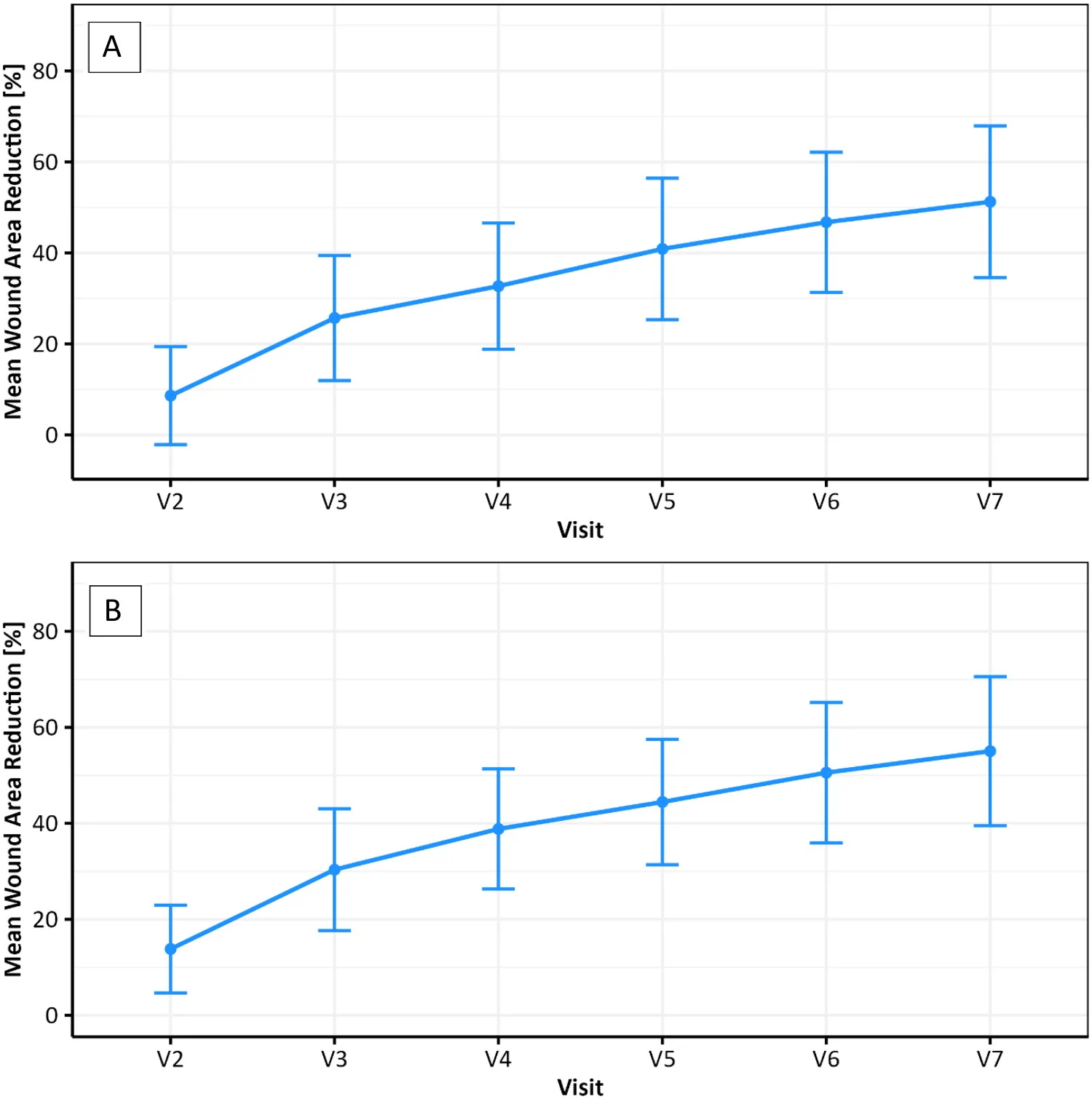
Mean percentage wound area reduction with 95% confidence intervals across visits 2–7. Panel A show measurements before cleansing/debridement; panel B shows measurements after cleansing/debridement.

### Patient-reported outcomes

Patient-reported outcome data were available for 52 patients at baseline and for 48 patients at the final/discontinuation visit; therefore, paired Wound-QoL and valid final PBI-W analyses were based on 48 patients (Supplementary Table S2). Four of these 52 patients did not have final/discontinuation PRO assessments available and were therefore excluded from paired Wound-QoL and final PBI-W analyses.

No ad hoc imputation was performed; item-level scoring rules were applied according to the instrument manuals.

For Wound-QoL-17, lower scores indicate less impairment. Global Wound-QoL improved from 2.03 ± 0.78 at baseline to 1.18 ± 0.80 at final/discontinuation visit. The mean improvement was 0.81 points (95% CI 0.49 to 1.12; paired t-test *p* < 0.001; Wilcoxon signed-rank *p* < 0.001), with a standardized response mean of 0.74. Clinically relevant improvement of at least 0.50 points in the global score was observed in 27 of 48 patients (56.3%); 16 (33.3%) were stable and 5 (10.4%) worsened by at least 0.50 points (Supplementary Table S3). Domain-level results are shown in Table 2, and item-level Wound-QoL analyses are reported in Supplementary Table S4.

**Table 2.** Wound-QoL-17 baseline-to-final/discontinuation change.

Outcome	Baseline mean ± SD	Final mean ± SD	Mean improvement (95% CI)	Sensitivity and response
Global Wound-QoL	2.03 ± 0.78	1.18 ± 0.80	0.81 (0.49 to 1.12)	t *p* < 0.001; Wilcoxon *p* < 0.001; SRM 0.74; MID improved 27/48 (56.3%)
Body subscale	1.63 ± 0.83	0.86 ± 0.79	0.71 (0.43 to 1.00)	t *p* < 0.001; Wilcoxon *p* < 0.001; SRM 0.72
Psyche subscale	2.46 ± 1.01	1.64 ± 1.05	0.78 (0.33 to 1.23)	t *p* < 0.001; Wilcoxon *p* = 0.001; SRM 0.51
Everyday-life subscale	1.88 ± 1.04	1.10 ± 0.90	0.74 (0.37 to 1.11)	t *p* < 0.001; Wilcoxon *p* < 0.001; SRM 0.58

Improvement is calculated as baseline minus final visit score; positive values indicate reduced impairment. Paired *N* = 48 for all domains.

Abbreviations. Wound QoL, Wound Quality of Life; SRM, standardized response mean.

PBI-W was analyzed as an attained patient-relevant treatment-benefit measure. Final PBI-W benefit was high, with a mean score of 2.92 ± 0.97 and median score of 3.07 (IQR 2.15 to 3.78) on the 0 to 4 scale (Table 3). PBI-W scores were at least 1 in 45 of 48 patients (93.8%), at least 2 in 42 of 48 (87.5%), and at least 3 in 28 of 48 (58.3%). Item-level PBI-W needs and benefit patterns, exploratory PBI-W sensitivity analyses, correlations among PRO measures and descriptive product subgroup summaries are reported in Supplementary Tables S5, S6, S7 and S8, respectively.

**Table 3.** Final attained patient benefit index for wounds (PBI-W) benefit.

Measure	*N*	Mean ± SD (95% CI)	Median (IQR)	Responder distribution
Final PBI-W attained benefit	48	2.92 ± 0.97 (2.63 to 3.20)	3.07 (2.15 to 3.78)	≥1: 45 (93.8%); ≥2: 42 (87.5%); ≥3: 28 (58.3%)

The PBI-W was interpreted as an attained treatment-benefit measure based on baseline treatment needs and follow-up goal attainment, not as a conventional change score. Scores range from 0 to 4; higher scores indicate greater patient-relevant treatment benefit.

### Social support and safety

Baseline perceived social support was high: the MSPSS total score was 5.74 ± 1.09 (median 6.00, IQR 5.00 to 6.67), with the strongest support from significant others (mean 6.11 ± 1.09) and family (mean 5.85 ± 1.44) (Supplementary Table S9). Exploratory correlation analyses supported convergence between the two patient-reported outcome measures: global Wound-QoL improvement was moderately correlated with final PBI-W attained benefit (Spearman rho = 0.42; *p* = 0.003), while baseline social support was not meaningfully associated with either Wound-QoL improvement or final PBI-W benefit (Supplementary Table S9). Safety findings were favorable. In the available study records, 10 adverse events were reported in 7 participants; consisting mainly of localized periwound skin reactions. No dressing-related serious adverse events were reported, and no withdrawals were attributed to the investigational dressings.

## Discussion

This VLU subgroup adds patient-level outcomes evidence to a field in which endpoint selection has historically been heterogeneous and often dominated by healing alone. The CoreVen scoping review showed substantial variability in VLU trial outcome reporting, and the subsequent international eDelphi core outcome set recommended that VLU evaluations routinely report healing, pain, quality of life, resource use, and adverse events, with the patient rather than the ulcer or limb as the preferred unit of analysis.^11^^,^^12^ The present focus on Wound-QoL-17 and PBI-W aligns with these priorities. The observed Wound-QoL results suggest clinically relevant change beyond statistical significance.

Baseline Wound-QoL scores in the present cohort indicated a considerable impact on patients' quality of life and are consistent with previous studies demonstrating that patients with VLU have a marked negative impact on quality of life. In a large community-based study using the Wound-QoL instrument, Smith et al.^47^ reported substantial quality-of-life impairment among patients with VLU, with 98% of participants reporting an adverse impact on quality of life. Differences in study populations and score reporting methods limit direct comparison of absolute Wound-QoL values.

Global Wound-QoL scores improved by 0.81 points from baseline to final visit, with a 95% confidence interval from 0.49 to 1.12, and 56.3% of paired patients achieved at least a 0.50-point improvement. Because a global Wound-QoL change of approximately 0.50 points has been proposed as a minimal important difference, the magnitude of improvement observed in this subgroup is compatible with clinically meaningful reduction in wound-related impairment.^39^ However, the lower bound of the 95% CI lies at the MID threshold, indicating compatibility with effects ranging from approximately minimal important change to nearly twice that magnitude. Although 56.3% of patients achieved the prespecified threshold for clinically meaningful improvement in global Wound-QoL, one-third remained stable and a small proportion worsened. Therefore, the findings should be interpreted as evidence of overall cohort-level improvement rather than benefit in all treated individuals.

These improvements are important because most of the domains that improved in this cohort map closely onto burdens repeatedly described by patients with VLU. Systematic reviews and qualitative syntheses show that pain, exudate, leakage, malodour, sleep disturbance, impaired mobility, social restriction, and psychological distress are major drivers of poor quality of life in venous ulceration.^4^^,^^5^^,^^48^ Exudate leakage and odour are particularly disruptive. Jones and colleagues linked these symptoms to embarrassment, disgust, low self-esteem, social isolation, and depression in people with chronic venous leg ulceration.^49^ These results support a patient-centered interpretation of treatment response beyond wound healing alone.

The use of Wound-QoL-17 is methodologically justified. Wound-QoL was developed from established chronic-wound quality-of-life instruments, has demonstrated feasibility and validity in chronic wounds, including leg ulcers.^17^^,^^18^^,^^38^ Wound-QoL remains a practical, wound-specific, prospectively collected legacy instrument with established scoring and interpretability, making it appropriate for this secondary VLU subgroup analysis.

PBI-W should be interpreted differently from Wound-QoL. PBI-W is a patient-defined attained-benefit measure derived from pre-treatment ratings of treatment needs and follow-up ratings of goal attainment.^19^ In this analysis, the mean attained PBI-W score was 2.92 on the 0–4 scale, indicating high patient-relevant benefit. This magnitude is externally plausible because an outpatient leg-ulcer pilot study reported a mean PBI-W score of 2.93 and found that patients valued confidence in diagnosis and therapy, rapid wound closure, pain reduction, and the ability to continue living normally.^20^ Wound-QoL-17 and PBI-W provide complementary perspectives on patient benefit. Wound-QoL captures longitudinal change in symptom burden and functional impairment, whereas PBI-W reflects goal attainment relative to patient-prioritized treatment needs. Together, these instruments provide a more comprehensive evaluation of treatment impact by integrating both standardized outcome measurement and individualized patient-defined benefit. The moderate association between lower final Wound-QoL burden and higher PBI-W benefit in the present cohort further supports convergence between reduced impairment and patient-defined benefit.

The present findings are also consistent with those reported by Armstrong et al. in the diabetic foot ulcer subgroup of the same parent prospective multicenter study.^50^ Although the wound populations differed, both analyses were associated with improvements in wound-related quality of life and favorable patient-perceived treatment benefit, supporting the value of incorporating patient-reported outcome measures alongside traditional clinical endpoints in chronic wound research. However, the findings should be interpreted with caution and viewed as hypothesis-generating real-world evidence, rather than definitive comparative proof of dressing superiority.

From a pharmacoeconomic perspective, the results are hypothesis-generating. VLU management entails substantial healthcare expenditure, and UK cost studies have identified community nursing time and repeated wound-care contacts as major cost drivers.^2^^,^^3^ Improvements in exudate control, patient comfort, and everyday-life burden could plausibly affect dressing-change burden and service use, but direct resource-use data were not collected prospectively for this subgroup. Accordingly, the present study supports the rationale for future comparative and economic evaluations of dressing strategies in VLU care but should not be presented as a formal economic analysis.

Several limitations should temper interpretation. This was an open-label, nonrandomized, single-arm subgroup analysis, and all patients received compression therapy, which is the cornerstone of VLU management and is associated with improved healing compared with no compression.^36^ The incremental contribution of the investigational dressings therefore cannot be disentangled from compression therapy, which is the standard of care and a major determinant of healing. In addition, regulatory and methodological PRO literature cautions that open-label designs can influence patient-reported responses because patient expectations and treatment knowledge may affect subjective assessment.^28^^,^^51^ The modest subgroup sample size, particularly within individual dressing subgroups, limited the ability to perform robust subgroup analyses. The 6-week follow-up, baseline-to-final PROM time structure, and absence of direct resource-use measurement further limit causal and economic inference. The relatively short 6-week follow-up period limited the assessment of complete wound healing, recurrence, and long-term patient-reported outcomes. The absence of a comparator group further limits attribution of observed effects to the intervention. Because a few patients discontinued before week 6, the timing of the final PRO measurement varied for some individuals (*n* = 4), which may introduce variability in the estimated magnitude of change; however, the impact is expected to be minimal given the limited number of early discontinuations. This study was conducted in Poland, which may limit generalizability to other healthcare systems and patient populations.

Despite these limitations, the analysis has several strengths. It uses prospectively collected patient-reported data, validated chronic-wound instruments, instrument-specific scoring rules, confidence intervals, sensitivity analyses, and a published minimal important difference to support clinically interpretable reporting. This approach is consistent with contemporary expectations for PRO reporting and patient-centered outcomes research, which emphasize clear PRO objectives, transparent instrument rationale, explicit scoring and missing-data handling, and interpretation beyond statistical significance.^26^^,^^29^^,^^33^^,^^35^^,^^42^

## Conclusion

In this VLU subgroup of a prospective nonrandomized cohort study, treatment with silicone superabsorbent polymer dressings under compression therapy was associated with statistically significant and patient-relevant improvement in wound-related quality of life. The observed mean global Wound-QoL-17 improvement exceeded the published minimal important difference, and more than half of patients with paired data achieved clinically relevant improvement. Follow-up PBI-W scores indicated favorable attained patient-defined treatment benefit, complementing the Wound-QoL findings by showing that benefits were meaningful in relation to patient-prioritized treatment goals.

These findings support the inclusion of validated wound-specific patient-reported outcome measures in VLU evaluations and reinforce the importance of assessing dressing performance from the patient perspective alongside conventional clinical endpoints. The findings are consistent with previous evidence demonstrating that VLUs are associated with a substantial quality-of-life burden and reinforce the value of incorporating patient-reported outcomes alongside conventional clinical endpoints. Furthermore, the observed improvements in patient-reported outcomes complement the favorable wound-healing outcomes observed in this cohort and support a more comprehensive assessment of treatment impact in chronic wound care.

Because the study was open-label, nonrandomized, single-arm, and conducted alongside standard compression therapy, the results cannot isolate the incremental effect of the dressing or establish cost-effectiveness. Future randomized or well-controlled real-world studies should combine clinical endpoints, wound-specific PROMs, adverse-event reporting, and prospective resource-use data to define the comparative and economic value of silicone superabsorbent dressing strategies in VLU care.

## Supplementary Material

VLU_PROM_supplementary_material_revised_V04.docx

## Data Availability

Study data supporting the findings of this subgroup analysis shall be made available according to sponsor policy and applicable ethics and privacy requirements.

## Transparency

### Use of artificial intelligence-assisted technology

The authors used Microsoft 365 CoPilot (Microsoft Corp., US) to review language, spelling and grammatical errors.
