# Supplementary material for: Patient-reported outcomes in venous leg ulcers: results from a prospective multicenter single-arm cohort study of silicone superabsorbent polymer dressings
Source: Patient Relat Outcome Meas. 2026 Oct 4;17(1):136–48. doi: 10.1080/1179271X.2026.2734965 (PMC13644622; doi:10.1080/1179271X.2026.2734965)
Supplement: Supplementary file 1 [file DPRO_A_2734965_SM5191.docx]

**Supplementary Material**

This supplementary document compiles source baseline tables and extended patient-reported outcome analyses used to prepare the revised manuscript. All patient-reported outcome analyses were exploratory and were not adjusted for multiplicity.

**Supplementary Table S1. Lifestyle and prior wound treatment before enrolment (overall VLU cohort)**

| Variable | Overall (N = 52) |
| --- | --- |
| Current smoking | 4 (7.7%) |
| Daily cigarettes among smokers, mean (SD) | 7.3 (8.7) |
| Systemic agents used before enrolment | 24 (46.2%) |
| Topical agents used before enrolment | 8 (15.4%) |
| Gauze used before enrolment | 15 (28.8%) |
| Foam dressings used before enrolment | 13 (25.0%) |
| Hydrocolloid dressings used before enrolment | 1 (1.9%) |
| Silicone dressings used before enrolment | 2 (3.8%) |

# Supplementary PRO analyses

Wound-Quality of Life (Wound-QoL) improvements are calculated as baseline minus final visit score, so positive values indicate reduced impairment. Patient Benefit Index – Wounds (PBI-W) is summarized as an attained patient-relevant benefit score and is not analyzed as a conventional baseline-change endpoint.

**Supplementary Table S2. PRO data availability**

| Measure | Baseline valid N | Final valid N | Paired/usable N |
| --- | --- | --- | --- |
| Global Wound-QoL | 52 | 48 | 48 |
| Body subscale | 52 | 48 | 48 |
| Psyche subscale | 52 | 48 | 48 |
| Everyday-life subscale | 52 | 48 | 48 |
| PBI-W final attained benefit | 52 | 48 | 48 |
| MSPSS baseline total score | 51 | — | — |

**Supplementary Table S3. Wound-QoL responder analysis using the 0.50-point threshold**

| Domain | Paired N | Improved ≥0.50, n (%) | Stable, n (%) | Worsened ≥0.50, n (%) |
| --- | --- | --- | --- | --- |
| Global Wound-QoL | 48 | 27 (56.3%) | 16 (33.3%) | 5 (10.4%) |
| Body subscale | 48 | 26 (54.2%) | 18 (37.5%) | 4 (8.3%) |
| Psyche subscale | 48 | 29 (60.4%) | 9 (18.8%) | 10 (20.8%) |
| Everyday-life subscale | 48 | 27 (56.3%) | 16 (33.3%) | 5 (10.4%) |
| Standalone item 17 | 48 | 32 (66.7%) | 11 (22.9%) | 5 (10.4%) |

The 0.50-point threshold is most appropriate for the global Wound-QoL score; subscale and item results are exploratory.

**Supplementary Table S4. Wound-QoL item-level analyses**

| Item | Construct | Baseline mean | Final mean | Mean improvement (95% CI) | Wilcoxon p | Improved ≥0.50 % |
| --- | --- | --- | --- | --- | --- | --- |
| 1 | Wound-related pain/discomfort | 1.96 | 0.77 | 1.15 (0.79 to 1.51) | <0.001 | 72.9% |
| 2 | Wound odour | 0.62 | 0.60 | -0.06 (-0.33 to 0.21) | 0.786 | 14.6% |
| 3 | Exudate/discharge burden | 2.04 | 0.92 | 1.08 (0.58 to 1.58) | <0.001 | 58.3% |
| 4 | Sleep affected | 1.38 | 0.73 | 0.60 (0.12 to 1.09) | 0.016 | 43.8% |
| 5 | Treatment burden | 2.17 | 1.29 | 0.79 (0.33 to 1.25) | 0.002 | 50.0% |
| 6 | Feeling unhappy | 1.96 | 1.54 | 0.38 (-0.12 to 0.87) | 0.115 | 52.1% |
| 7 | Feeling frustrated | 2.17 | 1.33 | 0.81 (0.32 to 1.31) | 0.003 | 56.3% |
| 8 | Worries about wound | 2.81 | 1.71 | 1.04 (0.52 to 1.56) | <0.001 | 64.6% |
| 9 | Fear of trauma to wound | 2.79 | 1.88 | 0.90 (0.39 to 1.40) | 0.002 | 66.7% |
| 10 | Fear of worsening | 2.56 | 1.73 | 0.77 (0.16 to 1.38) | 0.029 | 58.3% |
| 11 | Mobility restriction | 2.13 | 1.33 | 0.77 (0.33 to 1.22) | 0.001 | 58.3% |
| 12 | Leisure restriction | 1.88 | 1.10 | 0.74 (0.46 to 1.44) | <0.001 | 62.5% |
| 13 | Restriction in activities with others | 1.77 | 1.29 | 0.42 (-0.09 to 0.92) | 0.083 | 50.0% |
| 14 | Dependence on help | 2.08 | 1.09 | 0.91 (0.43 to 1.40) | <0.001 | 57.4% |
| 15 | Financial burden | 1.37 | 0.81 | 0.50 (0.04 to 0.96) | 0.039 | 37.5% |
| 16 | Problems with close relationships | 1.77 | 1.00 | 0.73 (0.30 to 1.16) | 0.002 | 50.0% |
| 17 | Overall wound burden | 2.71 | 0.92 | 1.83 (1.30 to 2.37) | <0.001 | 66.7% |

Item labels are shortened paraphrases. Positive improvement indicates lower final impairment than baseline.

**Supplementary Table S5. PBI-W attained benefit and exploratory sensitivity analyses**

| Measure | N | Mean ± SD | 95% CI | Median (IQR) | ≥1, n (%) | ≥2, n (%) | ≥3, n (%) |
| --- | --- | --- | --- | --- | --- | --- | --- |
| Final PBI-W attained benefit (baseline needs × final benefits) | 48 | 2.92 ± 0.97 | 2.63 to 3.20 | 3.07 (2.15 to 3.78) | 45 (93.8%) | 42 (87.5%) | 28 (58.3%) |
| Baseline same-visit PBI-W-like index (exploratory only) | 52 | 2.17 ± 1.13 | 1.86 to 2.49 | 2.07 (1.22 to 3.01) | 43 (82.7%) | 31 (59.6%) | 16 (30.8%) |
| Final same-visit PBI-W-like index (exploratory only) | 48 | 2.93 ± 0.97 | 2.65 to 3.21 | 3.07 (2.21 to 3.78) | 45 (93.8%) | 42 (87.5%) | 28 (58.3%) |

The primary PBI-W endpoint is the final attained benefit score based on baseline treatment needs and final benefit ratings. Same-visit PBI-W-like indices are provided only as exploratory sensitivity summaries

.

**Supplementary Table S6. PBI-W item-level treatment needs and benefit patterns**

| Item | Treatment goal | Need mean | Need quite/very % | Benefit mean | Benefit quite/very % | Mean need × benefit |
| --- | --- | --- | --- | --- | --- | --- |
| 1 | Freedom from pain | 3.65 | 89.8% | 3.22 | 78.3% | 12.09 |
| 2 | No wound drainage | 3.70 | 90.0% | 2.88 | 70.8% | 10.39 |
| 3 | No unpleasant wound smell | 3.11 | 72.2% | 2.89 | 63.9% | 9.00 |
| 4 | Wound healing | 3.80 | 94.1% | 2.79 | 59.6% | 10.70 |
| 5 | Better sleep | 3.24 | 78.6% | 2.90 | 73.8% | 9.36 |
| 6 | Less depression | 3.16 | 72.7% | 2.83 | 66.0% | 9.54 |
| 7 | Greater enjoyment of life | 3.53 | 86.7% | 2.83 | 63.0% | 10.32 |
| 8 | Less fear of worsening | 3.78 | 96.1% | 2.98 | 70.8% | 11.43 |
| 9 | Normal life | 3.65 | 89.8% | 2.89 | 66.0% | 10.78 |
| 10 | Productivity in everyday life | 3.73 | 93.9% | 2.98 | 70.2% | 11.31 |
| 11 | Not being a burden to family/friends | 3.58 | 86.7% | 3.00 | 71.1% | 11.25 |
| 12 | Normal spare time | 3.69 | 93.8% | 3.07 | 71.1% | 11.40 |
| 13 | Normal working life | 3.68 | 93.5% | 2.88 | 62.5% | 11.04 |
| 14 | More social contact | 3.46 | 87.0% | 2.94 | 66.0% | 10.63 |
| 15 | Comfort being out in public | 3.22 | 73.3% | 2.76 | 60.9% | 9.71 |
| 16 | Relationship with partner unaffected | 3.56 | 84.6% | 2.97 | 66.7% | 10.71 |
| 17 | Less reliance on doctor visits | 3.72 | 92.0% | 2.81 | 62.5% | 10.65 |
| 18 | Less time spent on wound care | 3.84 | 96.1% | 3.02 | 64.6% | 11.66 |
| 19 | Lower out-of-pocket treatment cost | 3.82 | 96.0% | 3.12 | 70.8% | 12.15 |
| 20 | Fewer side effects | 3.84 | 95.9% | 2.98 | 68.1% | 11.80 |
| 21 | Certainty about diagnosis and therapy | 3.88 | 97.9% | 3.00 | 68.8% | 11.43 |
| 22 | Confidence treatment will work | 3.78 | 95.9% | 2.98 | 68.1% | 11.07 |

Need and benefit scores range from 0 to 4; non-applicable items were excluded from item means.

**Supplementary Table S7. Exploratory correlations among PRO measures and baseline social support**

| Variable pair | N | Spearman ρ | Spearman p | Pearson r | Pearson p |
| --- | --- | --- | --- | --- | --- |
| Wound-QoL global improvement vs PBI-W final attained benefit | 48 | 0.42 | 0.003 | 0.44 | 0.002 |
| Wound-QoL baseline global score vs PBI-W final attained benefit | 48 | 0.14 | 0.337 | 0.16 | 0.265 |
| Wound-QoL final global score vs PBI-W final attained benefit | 48 | -0.46 | <0.001 | -0.46 | 0.001 |
| Baseline social support total vs Wound-QoL global improvement | 47 | -0.09 | 0.540 | 0.03 | 0.829 |
| Baseline social support total vs PBI-W final attained benefit | 47 | 0.05 | 0.732 | 0.14 | 0.336 |
| Wound-QoL psyche improvement vs PBI-W final attained benefit | 48 | 0.46 | <0.001 | 0.45 | 0.002 |
| Wound-QoL everyday-life improvement vs PBI-W final attained benefit | 48 | 0.36 | 0.012 | 0.33 | 0.022 |

Correlations were exploratory and were not adjusted for multiplicity.

**Supplementary Table S8. Descriptive PRO summaries by dressing type**

| Dressing | FAS N | Wound-QoL paired N | Wound-QoL mean improvement ± SD | Wound-QoL MID improved n (%) | PBI-W N | PBI-W mean ± SD | PBI-W median |
| --- | --- | --- | --- | --- | --- | --- | --- |
| RespoSorb Silicone Border | 34 | 30 | 0.78 ± 1.10 | 17 (56.7%) | 30 | 2.66 ± 1.02 | 2.97 |
| RespoSorb Silicone | 18 | 18 | 0.85 ± 1.10 | 10 (55.6%) | 18 | 3.34 ± 0.75 | 3.57 |

Dressing type was not randomized and was selected according to wound characteristics and clinical judgment; subgroup results are descriptive and hypothesis-generating only.

**Supplementary Table S9. Baseline perceived social support summary scores**

| Variable | Overall value |
| --- | --- |
| MSPSS total score, mean (SD) | 5.7 (1.1) |
| Significant other subscale, mean (SD) | 6.1 (1.1) |
| Family subscale, mean (SD) | 5.8 (1.4) |
| Friends subscale, mean (SD) | 5.2 (1.5) |
